# Serial measurement of pancreatic stone protein for the early detection of sepsis in intensive care unit patients: a prospective multicentric study

**DOI:** 10.1186/s13054-021-03576-8

**Published:** 2021-04-20

**Authors:** Jérôme Pugin, Thomas Daix, Jean-Luc Pagani, Davide Morri, Angelo Giacomucci, Pierre-François Dequin, Christophe Guitton, Yok-Ai Que, Gianluca Zani, David Brealey, Alain Lepape, Ben Creagh-Brown, Duncan Wyncoll, Daniela Silengo, Irina Irincheeva, Laurie Girard, Fabien Rebeaud, Iwan Maerki, Philippe Eggimann, Bruno François

**Affiliations:** 1 Service des soins intensifs, Hôpitaux Universitaires de Genève, Geneva, Switzerland; 2 Medical-Surgical Intensive Care Unit, Inserm CIC 1435 and UMR 1092, Dupuytren Teaching Hospital, Limoges, France; 3 Service of Intensive Care Medicine, Lausanne University Hospital and University of Lausanne, Lausanne, Switzerland; 4 Unità Operativa Anestesia e Rianimazione, Ospedale Infermi Rimini, AUSL della Romagna, Rimini, Italy; 5 Unità di Terapia Intensiva, Azienda Ospedaliera di Perugia, Perugia, Italy; 6 Médecine Intensive, Réanimation, Centre Hospitalier Régional Universitaire de Tours, Tours, France; 7 Service de Réanimation Médico Chirurgicale and USC, Centre hospitalier Le Mans, Le Mans, France; 8 Universitätsklinik für Intensivmedizin, Inselspital, Bern University Hospital, University of Bern, Bern, Switzerland; 9 Terapia Intensiva, Ospedale Santa Maria delle Croci, Ravenna, Italy; 10 Division of Critical Care and National Institute for Health Research University College London Hospitals Biomedical Research Centre, University College Hospital, London, UK; 11 Services de soins Critiques, Hôpital Lyon-Sud, Lyon, France; 12 Intensive Care Medicine, Royal Surrey County Hospital, Guildford, UK; 13 Department of Critical Care, Guy’s and St Thomas’ Hospital, London, UK; 14 Servizio Anestesia e Rianimazione, Ospedale San Giovanni Bosco, Turin, Italy; 15 Clinical Trial Unit (CTU) Bern, University of Bern, Bern, Switzerland; 16 Abionic SA, Lausanne, Switzerland; 17 Department of Locomotor System, Centre Hospitalier Universitaire Vaudois, Lausanne, Switzerland; 18 Réanimation Polyvalente, CHU Dupuytren, 2 avenue Martin Luther King, 87042 Limoges Cedex, France

**Keywords:** Pancreatic stone protein, Sepsis, Diagnostic, Procalcitonin, C-reactive protein, Biomarker

## Abstract

**Background:**

The early recognition and management of sepsis improves outcomes. Biomarkers may help in identifying earlier sub-clinical signs of sepsis. We explored the potential of serial measurements of C-reactive protein (CRP), procalcitonin (PCT) and pancreatic stone protein (PSP) for the early recognition of sepsis in patients hospitalized in the intensive care unit (ICU).

**Methods:**

This was a multicentric international prospective observational clinical study conducted in 14 ICUs in France, Switzerland, Italy, and the United Kingdom. Adult ICU patients at risk of nosocomial sepsis were included. A biomarker-blinded adjudication committee identified sepsis events and the days on which they began. The association of clinical sepsis diagnoses with the trajectories of PSP, CRP, and PCT in the 3 days preceding these diagnoses of sepsis were tested for markers of early sepsis detection. The performance of the biomarkers in sepsis diagnosis was assessed by receiver operating characteristic (ROC) analysis.

**Results:**

Of the 243 patients included, 53 developed nosocomial sepsis after a median of 6 days (interquartile range, 3–8 days). Clinical sepsis diagnosis was associated with an increase in biomarkers value over the 3 days preceding this diagnosis [PSP (*p* = 0.003), PCT (*p* = 0.025) and CRP (*p* = 0.009)]. PSP started to increase 5 days before the clinical diagnosis of sepsis, PCT 3 and CRP 2 days, respectively. The area under the ROC curve at the time of clinical sepsis was similar for all markers (PSP, 0.75; CRP, 0.77; PCT, 0.75).

**Conclusions:**

While the diagnostic accuracy of PSP, CRP and PCT for sepsis were similar in this cohort, serial PSP measurement demonstrated an increase of this marker the days preceding the onset of signs necessary to clinical diagnose sepsis. This observation justifies further evaluation of the potential clinical benefit of serial PSP measurement in the management of critically ill patients developing nosocomial sepsis.

*Trial registration* The study has been registered at ClinicalTrials.gov (no. NCT03474809), on March 16, 2018. https://www.clinicaltrials.gov/ct2/show/NCT03474809?term=NCT03474809&draw=2&rank=1.

**Supplementary Information:**

The online version contains supplementary material available at 10.1186/s13054-021-03576-8.

## Background

Sepsis, if not recognized and managed early, may evolve rapidly into life-threatening septic shock and multiple organ failure [1–3]. Sepsis and septic shock remain challenging global health problems associated with persistently high morbidity and mortality; in 2017, an estimated 48.9 million cases of sepsis were recorded worldwide, with 11.0 million related deaths, representing one-fifth of all causes of death [4]. Accordingly, guidelines systematically emphasize the early recognition and aggressive management of sepsis, with combined early antibiotic treatment and support to prevent organ dysfunction [1, 5, 6]. Currently, the confirmation of sepsis diagnosis is based largely on nonspecific clinical signs, laboratory findings and medical scores, which are usually obtained after sepsis onset. Despite extensive research, no biomarker has been identified with the capacity to detect sepsis quickly enough and with a high degree of diagnostic accuracy [7]. C-reactive protein (CRP) is a well characterized inflammatory marker widely used to help in the diagnosis of infection. Procalcitonin (PCT) has been extensively evaluated in the last 20 years as a marker of bacteremia. Even if CRP and PCT are commonly used in the context of the diagnosis of sepsis, both have shown suboptimal performance [8].

Pancreatic stone protein (PSP) is a C-type lectin protein that triggers polymorphonuclear cell activation and has shown proinflammatory activity in vitro [9]. In an unselected cohort of critically ill adults, PSP was found to be superior to PCT and other sepsis biomarkers for the accurate identification of sepsis [10]. An increase in PSP level preceding the development of sepsis has recently been demonstrated in a cohort of severely burnt patient [11]. The diagnostic performance of PSP, alone and in combination with other markers or clinical scores, was evaluated further in several studies conducted in adults, children, and neonates, in both, intensive care units (ICUs) and emergency departments [12]. In contrast to PCT and CRP, PSP was found to be a prognostic marker for ICU mortality [13–15]. In addition, point-of-care measurement of CRP and PCT is not common in ICU, while PSP can be measured with a ‘point-of-care’ device within 5 min using a single drop of whole blood [16], leading the way for simple, on-demand, around-the-clock, serial biomarker assessments instead of one-off measurements upon the clinical suspicion of sepsis.

We hypothesized that timely identification of changes in biomarker levels may help identify sepsis before the onset of clinical signs. We therefore designed a multicentric international prospective blind observational clinical study to explore the ability of serial PSP measurements to identify nosocomial sepsis before the onset of clinical signs required to clinically diagnose sepsis in an adult ICU population, and compare it with CRP and PCT.

## Study design and methods

### Study design

This multicentric biomarker-blinded prospective observational clinical study was conducted in 14 ICUs in France, Switzerland, Italy, and the United Kingdom, see Additional file 1: Table 1. Approval was obtained from the ethics committees of all participating sites. The study was registered at ClinicalTrials.gov (no. NCT03474809).

### Patient population

All patients older than 18 years old admitted to the ICU and expected to stay at least 7 days and/or to be mechanically ventilated for at least 5 consecutive days were screened for study inclusion (Additional file 1:Table 2). Patients with a clinical diagnosis of sepsis or suspicion of sepsis at the admission were not included. Written informed consent to participation and any study-related assessment was obtained from all patients before their inclusion in the study. Written informed consent was obtained according to the specific requirements of each center’s ethics committee.

At least 40 patients who developed sepsis after study enrolment were planned to be included. Based on recently published data [17–19], the expected incidence of sepsis in the targeted ICU population was estimated conservatively to be 15%. This value corresponded to a sample of 267 patients, to which we added 10% to compensate for withdrawal and loss to follow-up.

### Trial procedure and definitions

Patients were managed according to the centers’ standard clinical practices, including those for the diagnosis, assessment, and treatment of sepsis. Blood samples were collected daily for biomarker (PCT, CRP, and PSP) measurements. Patients were followed until death or discharge from the ICU or for 30 days, whichever occurred first. The Sepsis-3 criteria were used to define sepsis [1], and microbiological procedures were performed to diagnose infections responsible for sepsis according to local protocol.

### Measurement of biomarkers

Daily PSP levels were determined at the end of the study period with the CE-marked IVD PSP capsule on the point-of-care abioSCOPE® device (Abionic SA; Additional file 2: Figure 1). The 5th and 95th percentiles of PSP values in healthy adults are 25.0 to 60.7 ng/ml (median: 41.7 ng/ml) (Abionic internal data). CRP and PCT measurements were performed by a central laboratory using a Tina-quant® C-Reactive Protein Gen.3 (Roche Diagnostics) and a Liaison® Brahms PCT® for PCT (DiaSorin), respectively. All instruments were used according to the manufacturers’ instructions. The upper limit of the normal range of the CRP assay is 5 mg/l, and of the PCT assay 0.1 ng/ml, as per the manufacturer’s information.

### External adjudication committee

An independent endpoint adjudication committee (EAC) composed of three ICU experts was formed. Two non-chair EAC members retrospectively and independently reviewed case report forms for each patient and determined whether a septic event had occurred during the patient’s ICU stay (including on the day of inclusion), and on which day it started. Day 0 sepsis was determined using all information collected in the study’s eCRF and in accordance with the proposal published by Lambden et al. [20]. Discordant determinations were arbitrated by the EAC chair. The EAC had access to all case report forms, including results of all microbiological investigations, CRP and PCT measurements taken in the ICUs as part of standard care, but was blinded to centrally measured PSP, PCT, and CRP levels and the investigators’ diagnoses of sepsis.

### Patient-related data

Data collected at the time of inclusion were: demographic characteristics, reason for ICU admission, medical history, and Charlson comorbidity index. Additional clinical data were collected on day 1 and daily thereafter when measurements were performed as part of standard care; these data included vital signs (temperature, heart rate, and blood pressure), Sequential Organ Failure Assessment (SOFA) score, PaO_2_/FiO_2_ ratio, hematological data, clinical chemistry parameters, need for organ support (ventilation and use of vasoactive drugs) and microbiological data. In addition to the collection of data on antibiotic treatments (drug name, dose, schedule, route, and indication), meticulous assessments for sepsis detection were carried out daily until ICU discharge, death, withdrawal, or day 30, whichever came first.

### Statistical analyses

Discrete values are described as counts (percentages) and continuous variables are described as means with standard errors or medians with interquartile ranges (IQRs), as appropriate. The missing biomarker values were imputed by carrying the last observation forward, and multiple consecutive missing values were reported as missing. Based on EAC decisions, the patients were allocated to sepsis (a septic event occurred during the study period) or no-sepsis (no septic event during the study period) groups. In the sepsis group, the event day was set as the day on which the first septic events began, according to the EAC. In the no-sepsis group, the event day was set to day 7 of patients’ ICU stays, when more than 50% of adjudicated septic events occurred, or the days of discharge for patients with ICU stays of fewer than 7 days. Biomarkers average trajectories were visualized accordingly. All statistical analyses were performed using R version 4.0.2.

### Biomarkers dynamics and progression toward sepsis

To explore the dynamics of serial biomarker measurement throughout the study period, we modeled biomarker courses in the 3 days prior to EAC sepsis diagnoses by fitting a linear mixed-effect model with PSP, CRP, and PCT as response and the following explanatory variables: patient-specific random effect, group, day-to-event and group by day-to-event interaction as fixed-effects (estimates are provided in Additional file 1: Table 3). According to Van Breukelen [21] “testing absence of group by time interaction is equivalent to testing the hypothesis of no group effect on the change” in a response variable over days.

### Assessment of biomarker accuracy in diagnosing sepsis

The joint performance of the biomarkers in the diagnosis of sepsis on the day it occurred as per EAC decision was assessed using receiving operating characteristic (ROC) analysis. To account for differences among study sites and incidences of nosocomial sepsis, a linear mixed-effects logistic regression model was used with the center serving as a random intercept and biomarkers serving as fixed events to determine diagnostic accuracy, sensitivity, specificity, and the positive and negative likelihood ratios (estimates are provided in Additional file 1: Table 4) [22]. Cut-off values of PSP, CRP and PCT have been estimated without adjusting for center-effect.”

## Results

### Baseline characteristics of the study population

**Fig. 1:**
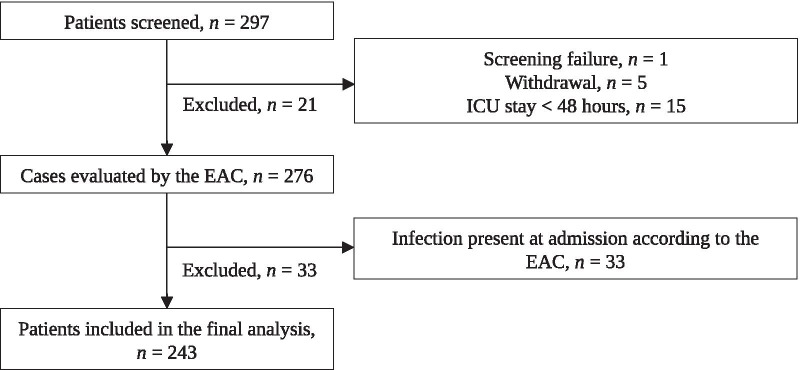
Flow of patient enrollment. There were 4 (0.14%) missing values for PSP (0; 0.0% after imputation), 8 (0.35%) for CRP (1; 0.04% after imputation) and 8 (0.28%) for PCT (2; 0.07% after imputation). *CRP* C-reactive protein, *EAC* endpoint adjudication committee, *ICU* intensive care unit, *PCT* procalcitonin, *PSP* pancreatic stone protein

From June 2018 to March 2019, 297 patients were enrolled in this study. The mean patients recruited per site was 21, and the median number was 15 (IQR: 8 to 23) (Additional file 1: Table 1b). Fifty-four of these patients were excluded from the analysis due to the presence of infection and/or sepsis at the time of inclusion as adjudicated by the EAC (*n* = 33), ICU stays < 48 h (*n* = 15), withdrawal (*n* = 5), and screening failure (*n* = 1) (Fig. 1). The characteristics of the remaining 243 patients are summarized in Table 1. The cohort contained 153 (63%) men and had a median age of 65 years (IQR: 54 to 73) and a median SOFA score of 6 (IQR: 5 to 9) at the time of admission. The most common comorbidities were cancer (16%) and heart (15%), pulmonary (13%), renal (12%), and hepatic (7%) diseases. Vasopressors were administered to 39% of patients, and 77% of patients required mechanical ventilation. The main reasons for ICU admission were acute central nervous system diseases (39%), cardiovascular diseases (31%), and abdominal/digestive conditions (19%; Table 1). The median ICU length of stay of the entire cohort was 9 days (IQR: 6 to 17); 7 days (IQR: 5 to 13 days) for the “no-sepsis” patients and 21 days for patients who developed sepsis (IQR: 14 to 27 days). ICU mortality rate in the study cohort was 16%. The baseline clinical characteristics of patients who did and did not develop sepsis during the study period were similar.

**Table 1:** Clinical characteristics of patients at the time of study admission (day 1 of intensive care unit stay) for the entire cohort and for the two sub-groups “sepsis” and “no-sepsis”

Variable	Modality/statistics	Total (*N* = 243)	No Sepsis (*N* = 190)	Sepsis (*N* = 53)	*p* value
Gender	F	90/243 (37%)	77/190 (41%)	13/53 (25%)	0.033^1^
	M	153/243 (63%)	113/190 (59%)	40/53 (75%)	
Age	Median (Q1;Q3)	65.0 (54.0;73.0)	65 (54;72)	64 (55;73)	0.915^2^
SOFA score	Median (Q1;Q3)	6 (5;9)	6 (4;9)	7 (5;9)	0.040^2^
Charlson score	0	51/243 (21%)	46/190 (24%)	5/53 (9%)	0.055^1^
	1–2	84/243 (35%)	60/190 (32%)	24/53 (45%)	
	3–4	47/243 (19%)	34/190 (18%)	13/53 (25%)	
	5 and more	52/243 (21%)	42/190 (22%)	10/53 (19%)	
	Non-reported Charlson score	9/243 (4%)	8/190 (4%)	1/53 (2%)	
Reason for ICU admission	CNS (trauma, stroke, haemorrhage)	94/243 (39%)	74/190 (39%)	20/53 (38%)	0.657^3^
	Cardiovascular	76/243 (31%)	60/190 (32%)	16/53 (30%)	
	Abdominal/digestive	45/243 (19%)	32/190 (17%)	13/53 (25%)	
	Respiratory	18/243 (7%)	16/190 (8%)	2/53 (4%)	
	Metabolic	9/243 (4%)	7/190 (4%)	2/53 (4%)	
ICU treatments	Mechanical ventilation	187/243 (77%)	143/190 (75%)	44/53 (83%)	0.236^1^
	Vasopressors	95/243 (39%)	69/190 (36%)	26/53 (49%)	0.175^1^
	No-reported vasopressors	16/243 (7%)	15/190 (8%)	1/53 (2%)	
Comorbidities	Cancer	40/243 (16%)	33/190 (17%)	7/53 (13%)	0.430^1^
	Heart disease	35/243 (14%)	26/190 (14%)	9/53 (17%)	0.590^1^
	Pulmonary disease	31/243 (13%)	26/190 (14%)	5/53 (9%)	0.381^1^
	Renal disease	29/243 (12%)	23/190 (12%)	6/53 (11%)	0.832^1^
	Liver disease	17/243 (7%)	12/190 (6%)	5/53 (9%)	0.543^3^
ICU length of stay	Median (Q1;Q3)	9 (6;17)	7 (5;13)	21 (14;27)	< 0.001^2^

The following tests were performed: ^1^Chi^2^; ^2^Wilcoxon; ^3^Fisher’s exact. Statistical significance was set a *p* value ≤ 0.05

*ICU* intensive care unit, *IQR* interquartile range, *SOFA* sequential organ failure assessment, *CNS* central nervous system

According to the EAC decisions, 21.8% (*n* = 53) of the patients developed sepsis and 78.2% (*n* = 190) did not. The EAC chair arbitrated disagreements about sepsis presence/absence and day of initiation in 31% of cases. Two thirds of them concerned the date of onset of the sepsis and were resolved by the chair of the committee. The median interval from study inclusion to sepsis development was 6 days (IQR: 3 to 8 days). The characteristics of patients who developed sepsis are provided in Table 2. Sepsis originated most frequently from respiratory tract infections (62%), followed by bloodstream infections (11%) and urinary tract (9%) infections.

**Table 2:** Characteristics of patients in the sepsis group at the time of endpoint adjudication committee diagnosis of sepsis

Characteristics	Sepsis
Number of patients (*n*)	53
Time interval of the clinical diagnosis of sepsis, median [IQR]	6 [3, 8]
SOFA score, median [IQR]	9 [6, 10]
ICU treatments (*n*, %)	
Mechanical ventilation	35 (66%)
Non reported mechanical ventilation	14 (26%)
Vasopressors	27 (51%)
Non reported vasopressors	1 (2%)
Biomarkers	
PSP, median (ng/ml) [IQR]	205 [120.3, 621]
CRP, median (mg/l) [IQR]	167.3 [77.8, 257.6]
PCT, median (ng/ml) [IQR]	0.77 [0.2, 2.2]
Site of infection (*n*, %)	47 (87%)
Respiratory tract (*n*, %)	29 (62%)
Bloodstream (*n*, %)	5 (11%)
Renal urinary tract (*n*, %)	4 (9%)
Abdominal (*n*, %)	3 (6%)
Other (*n*, %)	6 (13%)

*PSP* pancreatic stone protein, *CRP* C-reactive protein, *PCT* procalcitonin, *IQR* interquartile range, *ICU* intensive care unit, *SOFA* sequential organ failure assessment

### Time courses of biomarkers for sepsis prediction

**Fig. 2:**
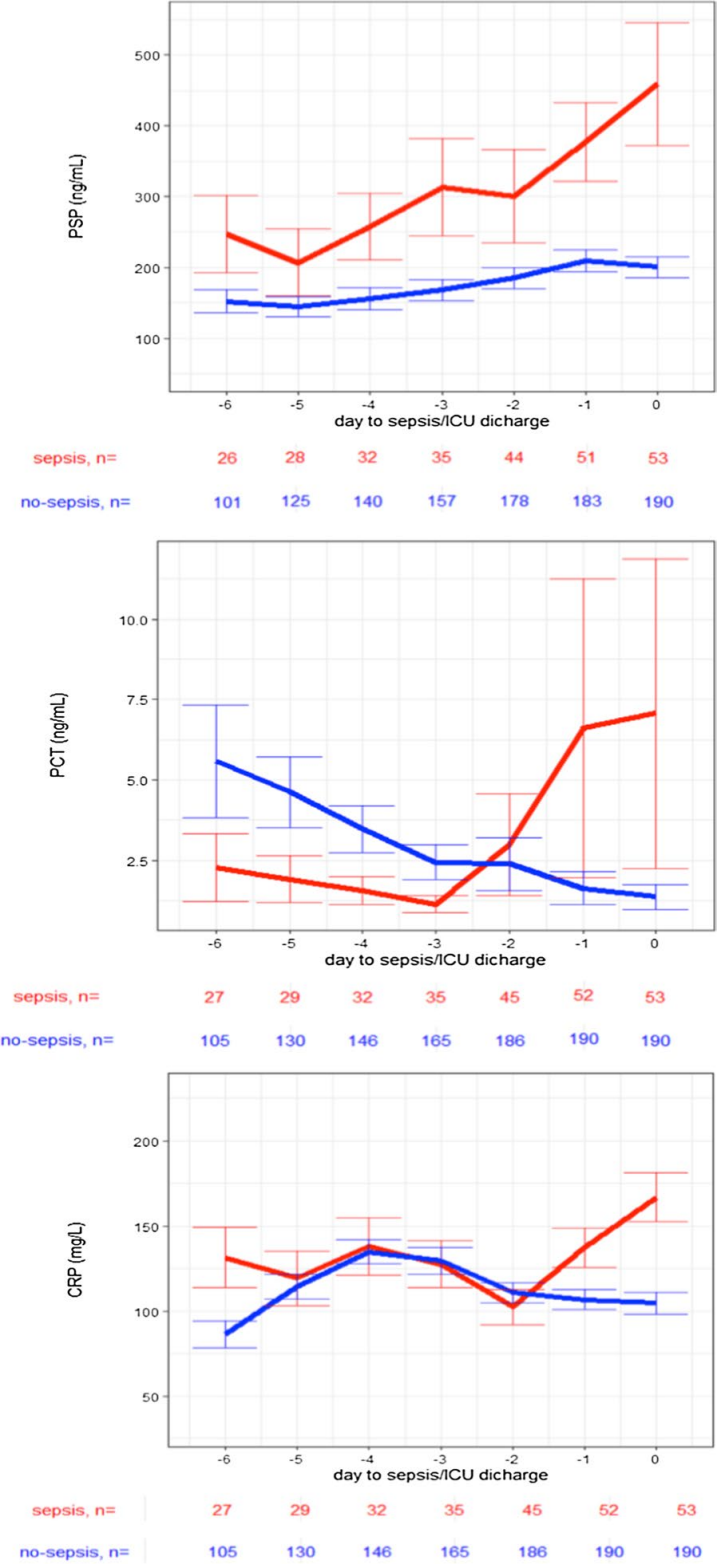
Association of pancreatic stone protein, procalcitonin and C-reactive protein increase with sepsis. Daily mean values ± standard errors of the mean for the sepsis group are from the 6 days preceding the endpoint adjudication committee diagnosis of sepsis. For the no-sepsis group, day 0 was set as intensive care unit day 7 or the day of discharge for intensive care unit stays < 7 days. The *p* values are for the association of clinical sepsis diagnosis with a continuous biomarker increase in the preceding 3 days (pancreatic stone protein, *p* = 0.003; procalcitonin, *p* = 0.025; C-reactive protein, *p* = 0.009). ns are numbers of observations per day and group. *PSP* pancreatic stone protein, *PCT* procalcitonin, *CRP* C-reactive protein, *EAC* endpoint adjudication committee, *ICU* intensive care unit

The time courses of biomarkers until sepsis (“sepsis” group), and until day 7 or discharge for ICU stays of fewer than 7 days (“no-sepsis” group) are shown in Fig. 2. All biomarker values increased for several days before the clinical diagnosis of sepsis (PSP, 5 days; PCT, 3 days; CRP, 2 days). Clinical sepsis diagnosis was associated with an increase in biomarkers over the 3 days preceding this diagnosis: PSP (*p* = 0.003), PCT (*p* = 0.025) and CRP (*p* = 0.009). Furthermore, estimates of exploratory variables are provided in the Additional file 1: Table 3.

### Accuracy of individual biomarkers and their combination in sepsis diagnosis

Areas under receiver operating characteristic curves (AUROCCs) on the day of sepsis diagnosis according to the blinded EAC were similar for all three biomarkers {PSP, 0.75 [95% confidence interval (CI) 0.67–0.82]; CRP, 0.77 [95% CI 0.69–0.84] PCT, 0.75 [95% CI 0.68–0.83]} (Table 3 and Additional file 3: Figure 2). The combined use of PSP with CRP improved the accuracy to 0.79 (95% CI 0.72–0.86). The addition of PCT to this combination did not further improve the accuracy or sensitivity, and only marginally improved the specificity. Estimates for the mixed-effects models for which the diagnostic performances are reported in Table 3 are provided in the Additional file 1: Table 4. Estimated cut-off values of biomarkers are 290.5 ng/ml for PSP, 167.2 mg/l for CRP and 0.94 ng/ml for PCT.

**Table 3:** Center-adjusted receiver operating characteristic results reflecting the ability of pancreatic stone protein, C-reactive protein, procalcitonin, and their combinations to discriminate the presence and absence of sepsis

Parameter	Accuracy	Sensitivity	Specificity	LR−	LR+
PSP	0.75 (0.67, 0.82)	0.74 (0.62, 0.86)	0.67 (0.60, 0.74)	0.40 (0.25, 0.63)	2.21 (1.70, 2.87)
CRP	0.77 (0.69, 0.84)	0.68 (0.55, 0.81)	0.77 (0.71, 0.83)	0.42 (0.28, 0.62)	2.91 (2.11, 4.02)
PCT	0.75 (0.68, 0.82)	0.72 (0.60, 0.84)	0.69 (0.62, 0.76)	0.41 (0.27, 0.64)	2.31 (1.75, 3.04)
PSP-CRP	0.79 (0.72, 0.86)	0.72 (0.60, 0.84)	0.73 (0.67, 0.80)	0.39 (0.25, 0.60)	2.69 (2.00, 3.61)
PSP-PCT	0.75 (0.68, 0.83)	0.76 (0.64, 0.87)	0.65 (0.58, 0.72)	0.38 (0.23, 0.61)	2.16 (1.68, 2.77)
CRP-PCT	0.77 (0.70, 0.84)	0.68 (0.55, 0.81)	0.77 (0.71, 0.83)	0.42 (0.28, 0.62)	2.91 (2.11, 4.02)
CRP-PCT-PSP	0.79 (0.72, 0.86)	0.72 (0.60, 0.84)	0.74 (0.68, 0.81)	0.38 (0.25, 0.59)	2.81 (2.08, 3.79)

*PSP* pancreatic stone protein, *CRP* C-reactive protein, *PCT* procalcitonin, *LR* likelihood ratio

## Discussion

This prospective multicentric study evaluated the potential of serial host protein biomarkers measurement, in particular the relatively new marker PSP, for the early identification of nosocomial sepsis in ICU patients. In the study cohort of critically ill patients at high risk of complications, in which one in five developed sepsis, the baseline clinical characteristics, including severity scores and the need for mechanical ventilation and vasopressor support, were similar among patients who did and did not develop sepsis. The main novel finding of this study is the significant association of the clinical diagnosis of sepsis with the continuous increase in the PSP level in the 3 days before this diagnosis. This association corroborates the initial description of this particularity of PSP [9], recently confirmed in a cohort of patients with severe burns, in which PSP levels increased constantly in the days preceding sepsis and this increase was greater in patients with septic shock [11, 23].

In addition, this study confirmed that the accuracy of clinical sepsis diagnosis based on PSP values was similar to that of diagnoses based on PCT and CRP values. Cut-off values for PSP the day of sepsis were however higher than usually reported in the literature, which is certainly due to the characteristics of the patients we included that differ from other studies with PSP in which it was evaluated at time of sepsis or suspected sepsis at admission. In an unselected population of critically ill patients, the use of PSP values to distinguish sepsis from non-infectious systemic inflammatory response syndrome (SIRS) at the time of ICU or high-dependency care admission was more accurate than the use of PCT values [10] was. Furthermore, a recent meta-analysis of data from 631 ICU patients, of whom 371 had infections or sepsis, confirmed that these conditions could be identified accurately based on PSP values [AUROCC = 0.81, slightly superior to those for PCT (0.78) and CRP (0.77)]. The combined use of PSP and CRP values increased this accuracy (AUROCC = 0.90) (Prazak J. et al., e-ISICEM, Sept. 15-18.2020), similarly to what was observed in this study where the combination of CRP and PSP increased the accuracy to 0.79, although the overlap of the 95% confidence intervals preclude to draw a robust statement regarding the significance of this increase.

This study has several strengths. First, it is the first international multicentric prospective study in which a host protein blood biomarker, the PSP, was measured daily in an ICU setting for the early detection of nosocomial sepsis using a point-of-care device offering on-demand test results in less than 10 min. Second, nosocomial sepsis was diagnosed by an independent adjudication committee composed of intensivists blinded to biomarker results. Third, this study determined the accuracy of single and combined biomarkers in the diagnosis of nosocomial sepsis in critically ill patients. Fourth, we explored the performance of the biomarkers, but we could not strictly interpret the value of bedside measurements of PSP compared to test performed in the clinical laboratory. However, several limitations of this study must be considered. Given its multicentric international design, the recruitment rate and sepsis incidence differed among study sites, in association with differences in preventive measures against nosocomial infection and sepsis development. In addition, although the expected number of patients who developed sepsis was reached, the low absolute number of sepsis events precluded subgroup analysis by site or country. Finally, this study was conducted with a subgroup of ICU patients who were expected to have prolonged ICU stays but were admitted free of infection, which precludes the extension of our observations to other settings, in particular for lower risk of developing nosocomial sepsis.

In the absence of an unambiguous definition of sepsis and highly accurate diagnostic tools the presence/absence of sepsis at any given days of an ICU stay was adjudicated by an expert committee. Discordant results using such approach have already been reported and vary considerably within sepsis diagnosis subgroups and clinical question [24], 25]. In our study not only the presence or absence of sepsis was adjudicated but also the day of event: this additional parameter contributed to the rate of discordance observed, reflecting the diagnostic challenges that clinicians are facing for the timely diagnosis of sepsis.

The inclusion of a risk-stratification of patients, for example based on clinical score such as the SOFA and the Acute Physiology And Chronic Health Evaluation (APACHE) scores, or of algorithms offering pretest probability in such diagnostic strategies, would allow to define economically viable and clinically meaningful strategies to closely monitor patients who will benefit the most of such approaches. In respect to that, the frequency of biomarkers measurement associated with the best clinical benefit remains to be established.

The clinical significance of an increase in the PSP level the days preceding the clinical diagnosis of sepsis may prompt changes in the management of patients at risk of nosocomial sepsis by triggering early diagnostic procedures and the timely establishment of appropriate treatment, for example to identify the source of the infection and the pathogen, and to assess the clinical utility of preemptive antibiotic therapy. Future interventional studies should also include a benefit-risk ratio of serial measurement of PSP, with a particular emphasis on the impact of false positive tests leading to unnecessary and potentially harmful diagnostic and therapeutic procedures. The results of this study may serve as a robust early basis for future validation studies of such an innovative approach, including studies conducted in wards outside of the ICU to determine how serial PSP measurement could enable early detection of sepsis before the onset of clinically overt symptoms in patients at risk.

## Conclusions

The diagnostic accuracy of PSP, CRP and PCT for the diagnosis of sepsis at the time the EAC identified it were similar. Serial measurements of biomarker revealed that blood PSP levels increased incrementally 3 days before the clinical diagnosis of nosocomial sepsis in critically ill patients, potentially allowing the early detection of sepsis before the appearance of signs and symptoms. These results justify further evaluation of the potential of serial PSP measurement in the early diagnosis, management and clinical outcome of critically ill patients developing nosocomial sepsis.

## Supplementary Information

**Additional file 1:** **Table 1.** (a) Study participating centers and (b) site recruitment. **Table 2.** Inclusion and exclusion criteria applied for patient recruitment. **Table 3.** Estimated coefficients of mixed-effects models for testing the consecutive increases in pancreatic stone protein, procalcitonin, and C-reactive protein levels serving as response variables. **Table 4.** Estimates for the mixed-effects models for which the results are reported in Table 3.

**Additional file 2: Figure 1.** The abioSCOPE® device and its in vitro diagnostic CAPSULE pancreatic stone protein.

**Additional file 3: Figure 2.** ROC curves for the diagnosis of sepsis at the time sepsis was clinically diagnosed by the EAC.

## Data Availability

All data generated or analyzed during this study are included in this published article and its additional files.

## Abbreviations

APACHE: Acute physiology and chronic health evaluation
AUROCC: Areas under receiver operating characteristic curves
CI: Confidence interval
CRP: C-reactive protein
EAC: External adjudication committee
ICU: Intensive care unit
IQR: Interquartile range
IVD: In vitro diagnostic
PCT: Procalcitonin
PSP: Pancreatic stone protein
SIRS: Systemic inflammatory response syndrome
SOFA: Sequential organ failure assessment
ROC: Receiver operating characteristic
