# Supplementary material for: Serial measurement of pancreatic stone protein for the early detection of sepsis in intensive care unit patients: a prospective multicentric study
Source: Crit Care. 2021 Apr 20;25:151. doi: 10.1186/s13054-021-03576-8 (PMC8056692; doi:10.1186/s13054-021-03576-8)
Supplement: Supplementary file 1 — Additional file 1: Table 1. (a) Study participating centers and (b) site recruitment. Table 2. Inclusion and exclusion criteria applied for patient recruitment. Table 3. Estimated coefficients of mixed-effects models for testing the consecutive increases in pancreatic stone protein, procalcitonin, and C-reactive protein levels serving as response variables. Table 4. Estimates for the mixed-effects models for which the results are reported in Table 3. [file 13054_2021_3576_MOESM1_ESM.docx]

*Additional Table 1a. Study participating centers*

| Participating centers | Cities | Country |
| --- | --- | --- |
| Centre Hospitalier Le Mans | Le Mans | France |
| Centre Hospitalier Universitaire Limoges | Limoges | France |
| Hôpital Bretonneau, Centre Hospitalier Régional Universitaire de Tours | Tours | France |
| Hôpital Lyon Sud, Centre Hospitalier Universitaire de Lyon | Pierre Benite | France |
| Ospedale Infermi Rimini | Rimini | Italy |
| Ospedale San Giovanni Bosco | Torino | Italy |
| Ospedale Santa Maria delle Croci | Ravenna | Italy |
| Santa Maria della Misericordia | Perugia | Italy |
| Bern University Hospital | Bern | Switzerland |
| centre hospitalier universitaire vaudois | Lausanne | Switzerland |
| Hopitaux Universitaires de Genève | Genève | Switzerland |
| Guy's & St Thomas' Hospital | London | United Kingdom |
| Royal Surrey County Hospital | Guildford | United Kingdom |
| University College London Hospitals | London | United Kingdom |

*Additional Table 1b. Site recruitment*

| **Site #** | **Recruited patients** |
| --- | --- |
| Site #1 | 80 |
| Site #2 | 49 |
| Site #3 | 37 |
| Site #4 | 23 |
| Site #5 | 22 |
| Site #6 | 20 |
| Site #7 | 15 |
| Site #8 | 14 |
| Site #9 | 10 |
| Site #10 | 8 |
| Site #11 | 8 |
| Site #12 | 7 |
| Site #13 | 3 |
| Site #14 | 1 |
| **Total: 14** | **297** |
| *Additional Table 1b: Number of patients included by site.* | |

*Additional Table 2. Inclusion and exclusion criteria applied for patient recruitment.*

| Inclusion criteria |
| --- |
| 1. Adult patients (age ≥ 18 years) |
| 2. Requiring ICU management |
| 3. At high risk of sepsis (septic patients are excluded) |
| 4. Expected ICU stay for at least 7 days or on mechanical ventilation for at least 5 days |
| 5. Have provided written informed consent or consent is given by the patient’s legally designated representative. |
| Exclusion Critera |
| 1. Patient expected to die within 48 hours of admission to ICU |
| 2. Patient on antibiotic treatment (a single antibiotic administration for surgical prophylaxis is allowed, as well as patients on antibiotic treatment but without signs, symptoms or any laboratory data suggesting a still active infection) |
| 3. Patient with chronic disease or prior medical history that will make some assessments unreadable (ie: uninterpretable chest X-ray film, etc) |
| 4. Patient suffering from or known acute or chronic pancreatitis, pancreatic cancer or admitted after pancreatectomy, but if a patient develops any pancreatic disease during the IUC stay he/she will remain in the study. |

*Additional Table 3. Estimated coefficients of mixed-effects models for testing the consecutive increases in pancreatic stone protein, procalcitonin, and C-reactive protein levels serving as response variables. .docx*

|  | PSP response | p-value | PCT response | p-value | CRP response | p-value |
| --- | --- | --- | --- | --- | --- | --- |
| Intercept | 223.4 (180.3, 266.4) | <0.001 | 1.0 (-1.3, 3.3) | 0.379 | 95.1 (80.0, 110.1) | <0.001 |
| Day to event | 11.0 (-0.9, 23.0) | 0.072 | -0.6 (-1.4, 0.1) | 0.086 | -9.5 (-15.0, -4.1) | 0.001 |
| Sepsis diagnosed by EAC (no sepsis is reference) | 214.0 (120.9, 307.1) | <0.001 | 6.3 (1.3, 11.2) | 0.014 | 41.3 (8.3, 74.3) | 0.015 |
| Interaction sepsis by day to event | 41.6 (13.9, 69.2) | 0.003 | 2.0 (0.3, 3.7) | 0.025 | 17.0 (4.3, 29.8) | 0.009 |
| # observations | 648 | | 673 | | 673 | |
| Standard deviation of random effect | 244.6 | | 11.8 | | 68.9 | |
| Residual standard deviation | 109.7 | | 6.9 | | 51.7 | |

*Additional Table 4. Estimates for the mixed-effects models for which the results are reported in Table 3.*

|  | 3 days to sepsis | p-value | 2 days to sepsis | p-value | 1 day to sepsis | p-value | sepsis day | p-value |
| --- | --- | --- | --- | --- | --- | --- | --- | --- |
| PSP estimates | | | | | | | | |
| Intercept | -1.96 (-2.72, -1.31) | <0.001 | -1.73 (-2.52, -1.05) | <0.001 | -1.78 (-2.46, -1.14) | <0.001 | -1.83 (-2.52, -1.21) | <0.001 |
| PSP effect | 0.002 (0.001, 0.004) | 0.011 | 0.001 (<0.001, 0.003) | 0.053 | 0.002 (0.001, 0.003) | 0.002 | 0.002 (0.001, 0.004) | <0.001 |
| Random effect standard deviation | 0.53 |  | 0.75 |  | 0.62 |  | 0.59 |  |
| # Observations | 192 |  | 222 |  | 234 |  | 233 |  |
| CRP estimates | | | | | | | | |
| Intercept | -1.47 (-2.28, -0.70) | 0.0001 | -1.21 (-2.08, -0.41) | 0.002 | -1.76 (-2.57, -1.02) | <0.001 | -2.04 (-2.82, -1.33) | <0.001 |
| CRP effect | <-0.001 (-0.004, 0.004) | 0.967 | -0.002 (-0.007, 0.003) | 0.418 | 0.004 (<0.001, 0.008) | 0.026 | 0.006 (0.003, 0.010) | <0.001 |
| Random effect standard deviation | 0.6 |  | 0.81 |  | 0.71 |  | 0.7 |  |
| # Observations | 192 |  | 222 |  | 234 |  | 233 |  |
| PCT estimates | | | | | | | | |
| Intercept | -1.39 (-2.05, -0.78) | <0.001 | -1.461 (-2.243, -0.803) | <0.001 | -1.28 (-1.92, -0.67) | <0.001 | -1.27 (-1.92, -0.67) | <0.001 |
| PCT effect | -0.061 (-0.270, 0.037) | 0.431 | 0.012 (-0.024, 0.041) | 0.385 | 0.019 (-0.001, 0.058) | 0.195 | 0.035 (0.002, 0.095) | 0.208 |
| Random effect standard deviation | 0.59 |  | 0.84 |  | 0.74 |  | 0.74 |  |
| # Observations | 192 |  | 222 |  | 234 |  | 233 |  |
