# Supplementary figures and images for: Serial measurement of pancreatic stone protein for the early detection of sepsis in intensive care unit patients: a prospective multicentric study
Source: Crit Care. 2021 Apr 20;25:151. doi: 10.1186/s13054-021-03576-8 (PMC8056692; doi:10.1186/s13054-021-03576-8)

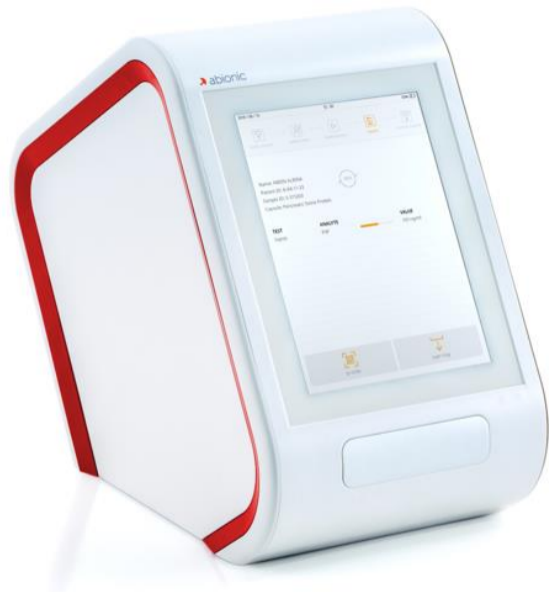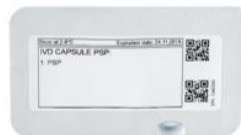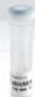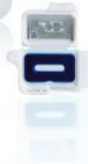

Supplement: Supplementary file 2 — Additional file 2: Figure 1. The abioSCOPE® device and its in vitro diagnostic CAPSULE pancreatic stone protein. [file 13054_2021_3576_MOESM2_ESM.pdf]
