# Supplementary material for: Serial measurement of pancreatic stone protein for the early detection of sepsis in intensive care unit patients: a prospective multicentric study
Source: Crit Care. 2021 Apr 20;25:151. doi: 10.1186/s13054-021-03576-8 (PMC8056692; doi:10.1186/s13054-021-03576-8)
Supplement: Supplementary file 3 — Additional file 3: Figure 2. ROC curves for the diagnosis of sepsis at the time sepsis was clinically diagnosed by the EAC. [file 13054_2021_3576_MOESM3_ESM.pdf]

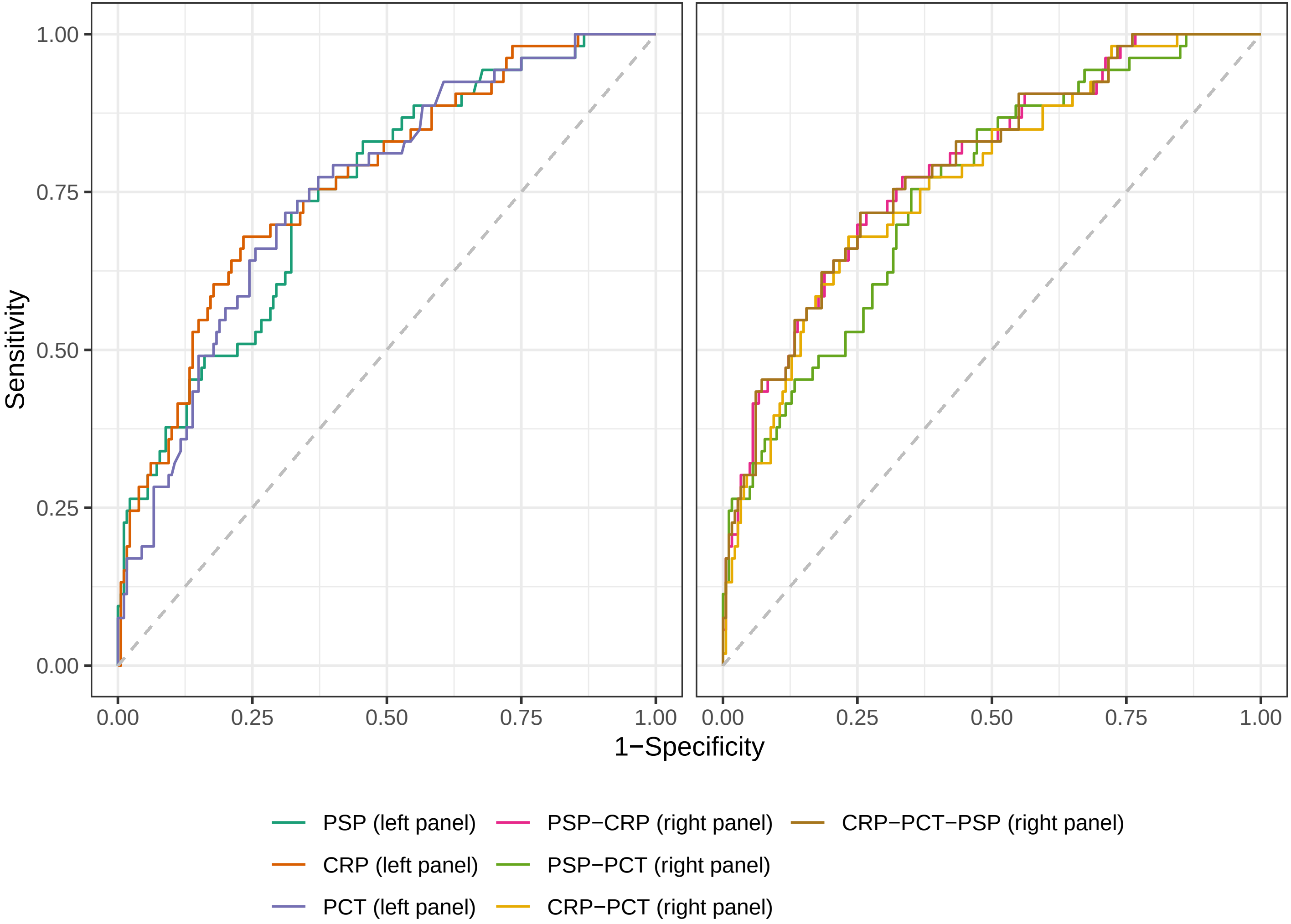

Sensitivity
1.00
0.75
0.50
0.25
0.00
0.00
0.25
0.50
0.75
1.00
0.00
0.25
0.50
0.75
1.00
1−Specificity
PSP (left panel)
CRP (left panel)
PCT (left panel)
PSP−CRP (right panel)
PSP−PCT (right panel)
CRP−PCT (right panel)
CRP−PCT−PSP (right panel)
